# Supplementary material for: Global, regional, and national burden and trends of kidney cancer associated with high BMI from 1990 to 2021: Findings from the Global Burden of Disease Study 2021
Source: PLoS One. 2025 Mar 24;20(3):e0320185. doi: 10.1371/journal.pone.0320185 (PMC11932458; doi:10.1371/journal.pone.0320185)
Supplement: Attachment — Submitted filename: Response to Reviewers.docx [file pone.0320185.s002.docx]

Dear Editors and Reviewers,

We appreciate the opportunity to revise our manuscript, "Global, regional, and national burden and trends of kidney cancer associated with high BMI from 1990 to 2021: findings from the Global Burden of Disease Study 2021" and thank the reviewers for their insightful comments. These opinions are very valuable for the revision and improvement of our paper, and also have important guiding significance for our research. We provide a detailed response to each reviewer's review. In addition, we have made comprehensive revisions to the entire manuscript and improved the picture quality. We have done our best to write all the revisions clearly and hope that the revised manuscript can meet the requirements for publication.

Sincerely,

Corresponding author.
